# Sulfate Attacks on Uncarbonated Fly Ash + Cement Pastes Partially Immersed in Na_2_SO_4_ Solution

**DOI:** 10.3390/ma13214920

**Published:** 2020-11-02

**Authors:** Zanqun Liu, Min Pei, Yuelin Li, Qiang Yuan

**Affiliations:** 1 School of Civil Engineering, National Engineering Laboratory for High Speed Railway Construction, Central South University, Changsha 410075, China; Zanqun.liu@csu.edu.cn (Z.L.); peimincsu@163.com (M.P.); 2 Department of Civil Engineering, KU Leuven, Campus Bruges, 8200 Bruges, Belgium

**Keywords:** chemical sulfate attack, physical sulfate attack, carbonation, cement

## Abstract

In this study, the sulfate attack on uncarbonated cement paste partially exposed to Na_2_SO_4_ solution was experimentally investigated and compared with that on carbonated specimens with the same exposure regime and uncarbonated specimens without exposure. N_2_ was used to protect specimens from carbonation throughout the sulfate exposure period. The effects of the water-to-cement (w/c) ratio and the fly ash as cement replacement on the sulfate attack were evaluated. Portland cement paste specimens with different w/c ratios of 0.35, 0.45, and 0.55 or fly ash replacement rates of 10%, 20%, and 30% were prepared. These specimens were partially immersed in 5% Na_2_SO_4_ solution for 50 d and 100 d exposure periods. The micro-analysis was conducted to evaluate the effect of the partial sulfate attack on the uncarbonated cement paste using X-ray diffraction (XRD) and thermo-gravimetric (TG) techniques. The results confirmed that, for uncarbonated cement paste, the chemical attack rather than the physical attack is the deterioration mechanism and is responsible for more severe damage in the evaporation zone (dry part) compared with the immersed zone (immersed part). When the effect of carbonation is well excluded, there is an optimal w/c ratio of 0.45 for minimizing the sulfate attack, while incorporating fly ash tends to reduce the sulfate attack resistance.

## 1. Introduction

When a concrete element is partially immersed in the sulfate-rich environment, the evaporation zone (or the part exposed to air) of the concrete generally deteriorates more severely than the immersion zone. In the past years, although many research works have been conducted to investigate the deterioration of the concrete partially exposed to sulfate attack [1,2,3,4], the controversy regarding the deterioration mechanism still exists. 

The sulfate attack mechanism can be classified into chemical attack and physical attack. The chemical sulfate attack means that, when the sulfate ions exist in or penetrate into cement-based materials, the chemical reactions between sulfates and cement paste tend to result in the generation of harmful gypsum, ettringite, or thaumasite, causing the expansion, cracking, scaling, and thus failure of the concrete [5]. The term physical attack (or salt weathering and crystallization distress) usually refers to the damage derived from the sulfates crystallization in the pores near the drying surface of porous materials (i.e., stone and masonry) partially exposed to the sulfate-rich environment, resulting in severe damage on the part exposed to air (or evaporation zone), but a sound part buried in the sulfate environment (or immersion zone) [6,7]. Physical sulfate attack is also attributed to concrete damage by the American Concrete Institute (ACI), as a similar damage phenomenon can be observed in the evaporation zone of concrete elements partially exposed to an environment rich in sulfate, especially Na_2_SO_4_ [8].

In the case of concrete partially exposed to sulfate attack, however, the theory of salt crystallization (or physical attack) cannot well explain the effect of several parameters, such as the water-to-cement (w/c) ratio and pozzolanic material, on the damage caused by sulfate attack. For example, it was found that the pozzolanic material addition tends to cause a more severe deterioration of concrete partially exposed to the sulfate environment [4,9]. The authors attributed this to the pore size refinement by the pozzolanic material, leading to an increase in capillary suction height and larger crystallization distress. Based on this mechanism, it can be expected that concrete with a lower w/c ratio should be more susceptible to physical attack than that with a higher w/c ratio, as reducing the w/c ratio can also refine the pore size. However, contrary results were found in the work by Nehdi and Hayek [10], where concrete with a w/c ratio of 0.45 showed more severe damage than that with a w/c ratio of 0.6. The conflicting data and the contradictory theory indicate that it seems extremely simplistic to only attribute the physical attack to the damage caused.

On the other hand, several research works [11] reported that chemical sulfate attack products (i.e., ettringite and gypsum) were identified in the evaporation zone of concrete partially exposed to sulfate attack, but no trace of sulfate crystals was detected. This indicates that the chemical attack could still be the deterioration mechanism of concrete partially exposed to sulfate attack. However, it is unreasonable to completely exclude the role of physical attack on damage caused, as many previous field cases have already confirmed the occurrence of physical attack [12]. This motivates researchers to explore the underlying conditions for damage caused by chemical and/or physical attacks.

The sulfate attack mechanism on concrete would be associated with the present Ca(OH)_2_ derived from cement hydration, as Ca(OH)_2_ could be a key source of the chemical sulfate attack: (1) Ca(OH)_2_ reacts with sulfates and Al ions to form gypsum and ettringite, resulting in expansion; (2) Ca(OH)_2_ concentration influences the pH value of the pore solution, and thus controls the stability of ettringite and C-S-H [13,14]. To check the role of Ca(OH)_2_ in the sulfate attack mechanism, a comparative study on damage regimes of Portland cement and calcium sulfoaluminate (CSA) cement partially exposed to sulfate attack was conducted [15]. The CSA cement, which has hydration products similar to Portland cement, but no Ca(OH)_2_, was used a reference sample (without Ca(OH)_2_) to examine the effect of Ca(OH)_2_. The results showed that CSA samples (absent of Ca(OH)_2_) exhibited physical sulfate attack damage, while Portland cement samples (containing Ca(OH)_2_) did not show any trace of sulfate crystallization, but generated chemical sulfate attack products. The physicochemical analysis indicated a potential mechanism: the absence of Ca(OH)_2_ could facilitate the physical attack, but prevent the chemical attack, owing to the disappearance of chemical reactions between Ca(OH)_2_ and sulfates [15].

Carbonation can cause the consumption of Ca(OH)_2_ in the carbonated area, and thus is expected to govern the deterioration mechanisms of sulfate attack by promoting the physical attack rather than chemical attack for carbonated concrete. A previous work by the authors examined the effect of carbonation on the physical sulfate attack on concrete in the evaporation zone [16]. Concrete specimens were acceleratingly carbonated for different periods to ensure different carbonation depths, and then partially exposed to 10% Na_2_SO_4_ solution. The results showed that, after the exposure, a larger carbonation depth caused a great concrete scaling due to the Na_2_SO_4_ crystallization. Based on these results, a viewpoint could be proposed that the physical attack could only occur in the carbonated area, while chemical attack still dominates the damage mechanism before carbonation.

To further verify this viewpoint, it is necessary to investigate deterioration mechanisms of the uncarbonated concrete partially exposed to the sulfate attack. This, however, has not been studied up to now, because the carbonation of concrete can hardly be prevented in both indoor and field tests owing to the presence of CO_2_ in air. To fill this gap, in this paper, the sulfate attack on different zones of uncarbonated cement paste partially exposed to Na_2_SO_4_ solution was experimentally investigated, and the specimens were protected by N_2_ to prevent carbonation throughout the exposure period. Moreover, owing to the exclusion of the effect of carbonation, the influence of the w/c ratio and the pozzolanic material (fly ash) on sulfate exposure was fundamentally investigated.

## 2. Materials and Methods 

### 2.1. Raw Materials 

In this study, P·I (42.5) cement and class-I fly ash meeting the requirements of GB175-2007 and GB/T 51003 were used. Table 1 gives the chemical compositions of both the cement and fly ash. An analytical reagent Na_2_SO_4_ was used to make a 5% Na_2_SO_4_ solution by mass.

### 2.2. Specimen Preparation and Curing 

The mix proportions of Portland cement paste samples and corresponding exposure periods are summarized in Table 2, where tap water was used for all mixtures. As shown in this table, PC0.35, PC0.45, and PC0.55 refer to paste made with water-to-cement ratios of 0.35, 0.45, and 0.55, respectively; and FA10, FA20, and FA30 refer to samples with fly ash as partial cement replacement by mass ratios of 10%, 20%, and 30%, respectively. For each mix proportion, 12 replicated specimens were made, among which 6 specimens were for 50-day exposure, while the rest were for 100-day exposure. Fresh pastes were cast in molds with the size of 10 mm × 40 mm × 150 mm, and molds were then sealed by plastic films to avoid moisture loss and carbonation. The specimens were demolded 24 h after casting and cured in saturated lime water at 20 ± 2 °C for 56 days, after which the specimens were removed from the solution and vacuum dried with silica gel for 7 days. Finally, the specimens were moved into a newly designed setup for partially sulfate exposure in the environment without CO_2_. The setup and exposure program will be described in detail in the following section.

### 2.3. Partial Sulfate Exposure

**Figure 1:**
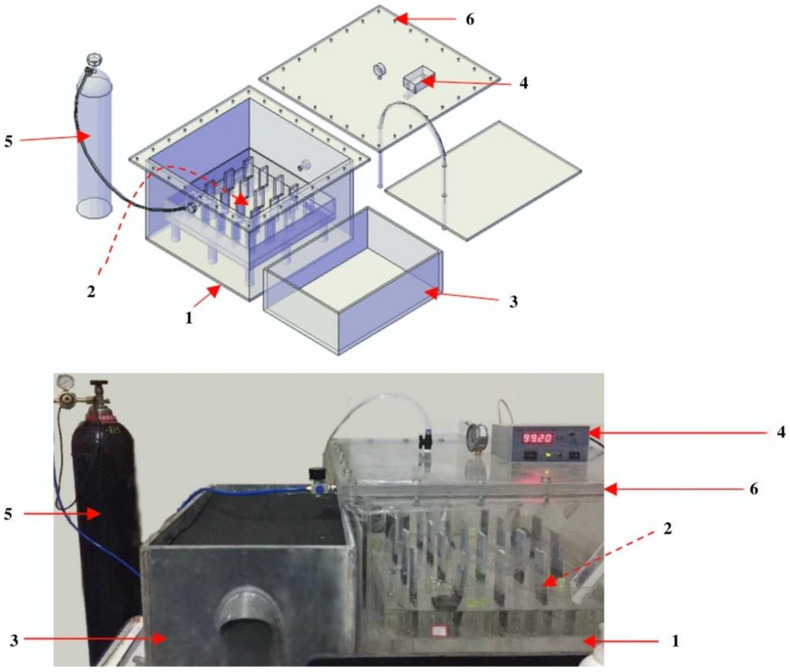
Schematic diagram and picture of the test set. 1—sealed chamber; 2—specimen box; 3—solution chamber; 4—nitrogen detector; 5—liquid nitrogen tank; 6—cover.

To prevent the specimens from carbonation, a new test setup for partially sulfate exposure was designed, as illustrated in Figure 1. The setup mainly consists of three containers:

1—Sealed chamber: a box with a 6—Cover contains a certain amount of saturated MgCl_2_ solution. The solution is used to ensure the humidity in the sealed chamber; with the evaporation of water in the solution, the humidity in the sealed chamber will increase gradually and finally maintain the humidity at 65% ± 5%. N_2_ is slowly poured into the sealed chamber to maintain an N_2_ concentration above 95% throughout the experiment.

2—Specimen box: an open box with a height of 40 mm containing the 5% Na_2_SO_4_ solution, in which the paste specimens are partially exposed in the solution with an immersion height of 30 mm. In this box, a plate with 20 rectangular holes with a dimension of 10 mm × 40 mm is used to support the specimens. The 2—Specimen box is supported by nine columns with a height of 60 mm from the bottom of the 1—Sealed chamber.

3—Solution container: an open box with a cover containing the fresh 5% Na_2_SO_4_ solution. The solution level in the 2—Specimen box is constantly maintained using a connecting vessel to 3—Solution container.

An additional six samples of PC0.45 were made, of which the upper part was exposed to air for carbonation, while the lower part was immersed in 5% Na_2_SO_4_ solution for 100 days, in order to confirm the effect of carbonation on the sulfate attack of the evaporation zone.

### 2.4. Micro-Analysis

Typical samples collected from the evaporation and immersion zones of the specimens after 50 days and 100 days of the sulfate exposure were collected for micro-analysis. Samples were oven-dried at 50 °C for 48 h and then grounded into powder smaller than 75 μm before the test. X-ray diffraction (XRD) tests were performed using a Bruker D8 Advance diffractometer from Bruker AXS GmbH, Karlsruhe, Germany (Cu Kα radiation, 30 mA, 40 kV) with a super speed detector. The measurements were carried out in a 2θ range of 5–65° with a step width of 0.025° and 0.5 s counting time per step. Thermogravimetric (TG) tests were carried out with a TGA 2(SF) for Mettler Toledo, Columbus, OH, USA. Approximately 50 mg of the prepared powder samples was tested from 30 °C to 800 °C at a heating rate of 10 °C/min under an N_2_ atmosphere.

## 3. Results

### 3.1. Visual Observation

#### 3.1.1. Effect of Carbonation 

**Figure 2:**
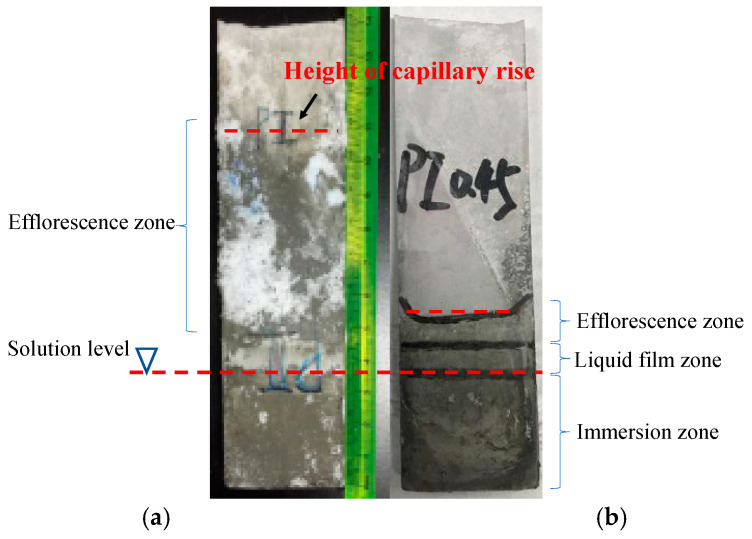
Visual observation on carbonated (**a**) and uncarbonated (**b**) specimens of PC0.45 after 100 days of partial sulfate attack.

Figure 2 presents the visual appearance of carbonated (in the air environment) and uncarbonated (in N_2_ environment) specimens after the sulfate exposure. The significant carbonation has occurred in specimens exposed to the air environment, which will be verified later by the X-ray diffraction (XRD) analysis.

For the evaporation zone (the part exposed to air), a proportion of this zone in the state of surface moisture was observed in both carbonated and uncarbonated specimens. This indicates the penetration of the sulfate solution into the evaporation zone by capillary suction. However, a higher capillary rise was found in the carbonated specimen than in the uncarbonated specimen. Moreover, an enormous amount of salt efflorescence was formed in the evaporation zone of the carbonated specimen, whereas almost no salt crystals appeared on the surface of the uncarbonated specimen. On the other hand, no significant difference in visual observation can be seen in the immersion zone of the two specimens.

It is well known that the carbonation of the cement-based materials reduces the porosity and the diameter of pores [16]. This could be the reason for the higher capillary suction of the carbonated specimen, and thus a larger salt efflorescence area. It is worth noting, however, that the larger amount of the salt efflorescence on the surface of the carbonated specimen cannot automatically mean that the carbonation can promote the deterioration by the physical attack. In fact, internal crystallization (or sub-efflorescence) instead of efflorescence on the surface is responsible for the deterioration by the physical attack [17]. In other words, the efflorescence on the surface causes no damage to the interior of the material. On the other hand, for the uncarbonated specimen, the presence of sulfate crystals appeared on the surface. This suggests that sulfates were either crystalized internally, resulting in harmful sub-efflorescence (or physical attack), or reacted with portlandite and other hydration products to form harmful erosion products, such as gypsum and ettringite. Therefore, internal chemical components of carbonated and uncarbonated specimens after sulfate exposure will be characterized to reveal how the absence of carbonation affects the sulfate attack deterioration and corresponding mechanisms.

#### 3.1.2. Effect of w/c Ratio and Fly Ash Replacement 

**Figure 3:**
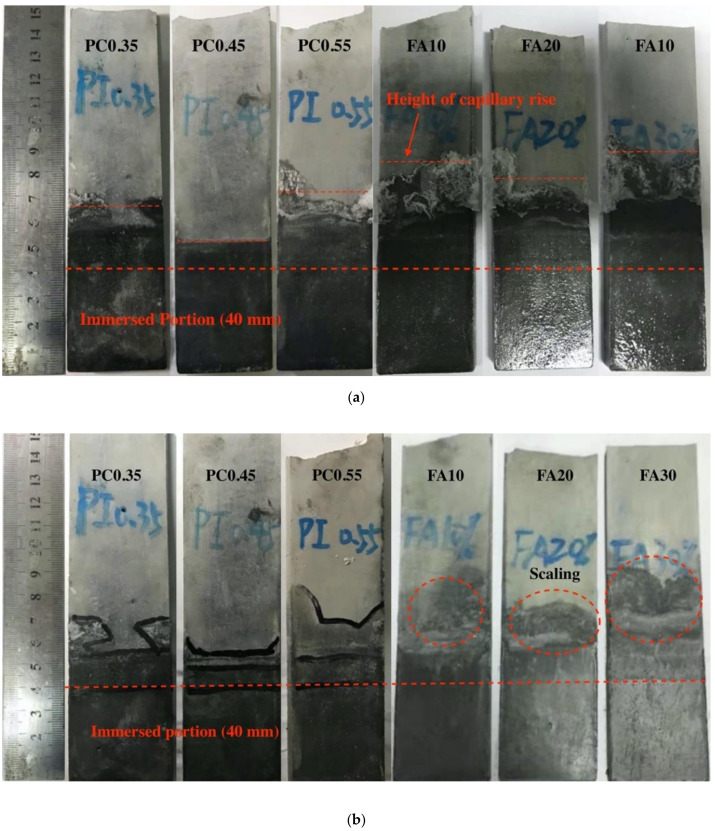
Visual observation on uncarbonated specimens with different w/c ratios and fly ash replacement rates after 100 days of partial sulfate exposure (**a**) after exposure and (**b**) after the crystals were removed.

The visual appearance of uncarbonated specimens with different w/c ratios and fly ash replacement rates after the sulfates exposure is given in Figure 3. As shown in Figure 3a, all specimens showed some white crystals attached on the surface of the evaporation zone, except for PC0.45, where no obvious crystals formed. In contrast, no significant damage can be found in the immersion zone for all specimens by visual observation.

Figure 3b presents the visual appearance of specimens after the crystals in the evaporation zone were removed. In case of the specimens with different w/c ratios, the evaporation zones were relatively intact for all specimens. However, it is interesting that, with the increase in w/c ratios, the height of capillary suction was first decreased and then increased, and the lowest value was seen in PC0.45. This indicates that a w/c ratio of 0.45 could provide the best sulfate resistance for the evaporation zone, as the lowest amounts of sulfates could enter the evaporation zone. On the other hand, for the specimens incorporated fly ash, the surface scaling was observed, and a significant higher capillary rise was found compared with the reference specimen PC0.45. This indicates the fly ash replacement tends to reduce the sulfate resistance in the evaporation zone. The micro-analysis will be conducted to examine the potential mechanism for the deterioration.

### 3.2. XRD Characterization on Chemical Components 

#### 3.2.1. Effects of Sulfate Exposure

**Figure 4:**
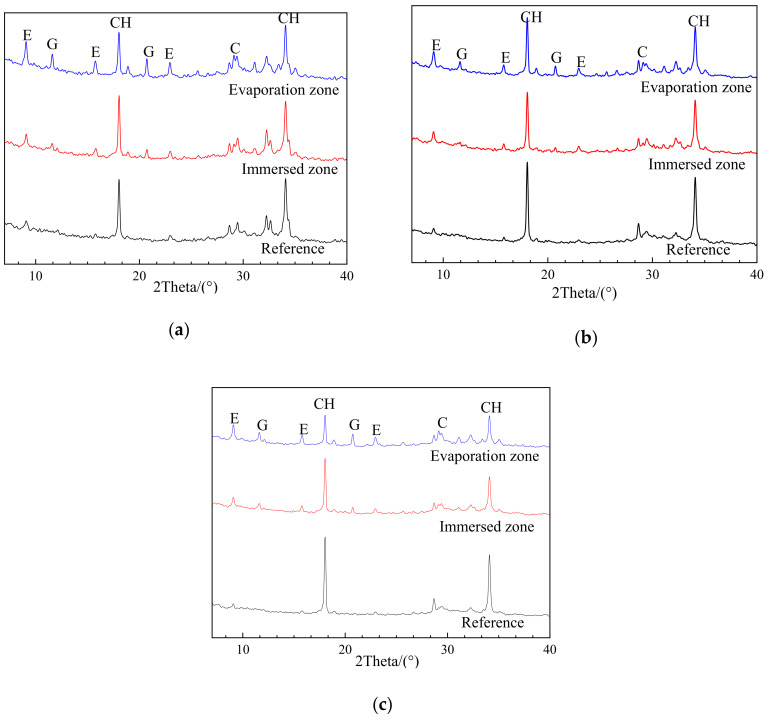
X-ray diffraction (XRD) patterns of (**a**) PC0.35, (**b**) PC0.45, and (**c**) PC0.55 after 100 days of sulfate exposure in N_2_ environment (reference—without erosion; E—Ettringite; G—CaSO_4_ × 2H_2_O; CH—Ca(OH)_2_; C—CaCO_3_).

**Figure 5:**
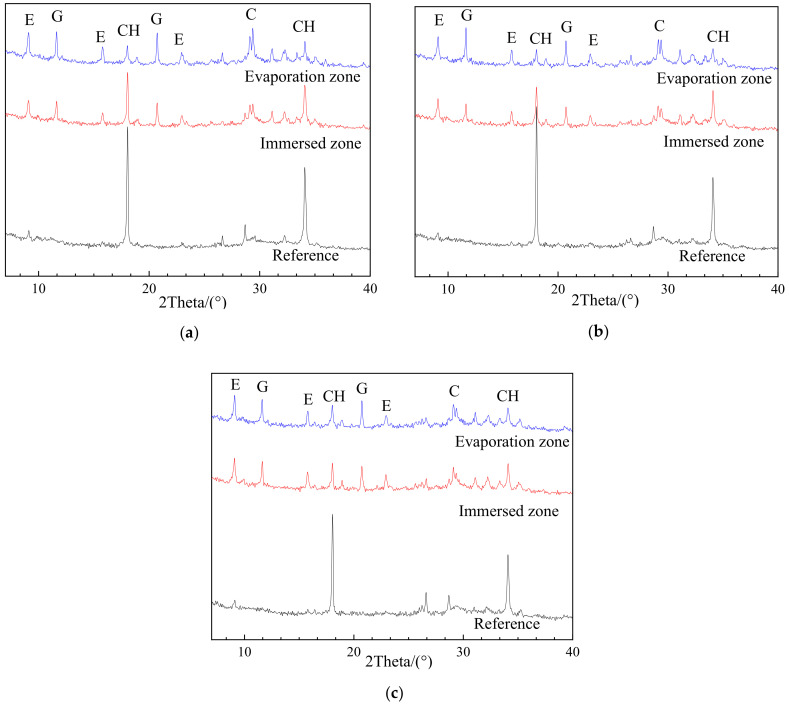
XRD patterns of (**a**) FA10, (**b**) FA20, and (**c**) FA 20 after 100 days of sulfate exposure in N_2_ environment (reference—without erosion; E—Ettingite; G—CaSO_4_ × 2H_2_O; CH—Ca(OH)_2_; C—CaCO_3_).

The XRD patterns of uncarbonated specimens with different w/c and fly ash as cement replacement after sulfate exposure, as well as corresponding reference samples without sulfate exposure, are given in Figure 4 and Figure 5. The upper parts of those specimens were either protected by N_2_ or sealed by plastic films to avoid carbonation.

As shown in Figure 4 and Figure 5, all the reference samples without sulfate exposure, regardless of w/c ratio and fly ash replacement rates, have no trace of gypsum, and an insignificant amount of ettringite likely to be derived from cement hydration. After exposure, however, the gypsum was easily identified and quite strong peaks of ettringite can be observed in the evaporation zones of all the specimens. These findings are powerful evidence that the chemical attack occurred in the evaporation zone after sulfate exposure. The chemical sulfate attack on the evaporation zone was ascribed to the sulfate ions migrating from the immersion to the evaporation zone due to capillary suction, followed by their reaction with the cement hydration products to generate chemical attack products [15]. On the other hand, the partial sulfate exposure caused no physical attack on the evaporation zone, because no traces of sulfate crystallization were detected by XRD analysis, as mentioned earlier. As a result, it can be concluded that the chemical sulfate attack rather than the physical sulfate attack is the deterioration mechanism for the evaporation zone of the uncarbonated cement partially exposed to the sulfate attack.

#### 3.2.2. Effects of Exposure Zone

As shown in Figure 4 and Figure 5, when compared with chemical components of the reference sample (without exposure), corrosion products such as gypsum were generally formed in both the evaporation and immersion zone of samples exposed to sulfate attack. This suggests that the chemical attack is responsible for the deterioration of both the evaporation and immersion zone of uncarbonated specimens. On the other hand, the chemical components in the evaporation and immersion zone were strongly influenced by the w/c ratio and the fly ash replacement.

In the case of uncarbonated specimens with different w/c ratios, as shown in Figure 4, there are significant differences in XRD patterns between the evaporation zone and the immersion zone. For example, generally, the corrosion products, especially gypsum, can hardly be detected in the immersion zone of all specimens, while significant amounts of gypsum and ettringite were identified in the evaporation zone. This phenomenon is particularly obvious in PC0.45, where almost no gypsum can be observed in the immersion zone, implying a negligible chemical attack. These results indicate that a more severe (chemical) sulfate attack could occur in the evaporation zone than in the immersion zone, and that the w/c ratio of 0.45 could provide better sulfate attack resistance when compared with the w/c ratio of 0.35 and 0.55.

In contrast, the phenomenon mentioned above was not found in uncarbonated specimens incorporated fly ash, as shown in Figure 5. For example, the chemical components of those specimens characterized by XRD in the evaporation zone and the immersion zone were comparable, and a certain amount of corrosion products (i.e., gypsum and ettringite) was formed in both zones. As mentioned earlier, the immersion zone of specimens with a w/c ratio of 0.45 showed a negligible sulfate attack. However, when incorporated fly ash, the specimens exhibited obvious sulfate attack in the immersion zone, reflected by the formation of corrosion products. These findings suggest that the fly ash addition could accelerate the chemical attack on cement.

#### 3.2.3. Effects of Carbonation on Deterioration Regime

**Figure 6:**
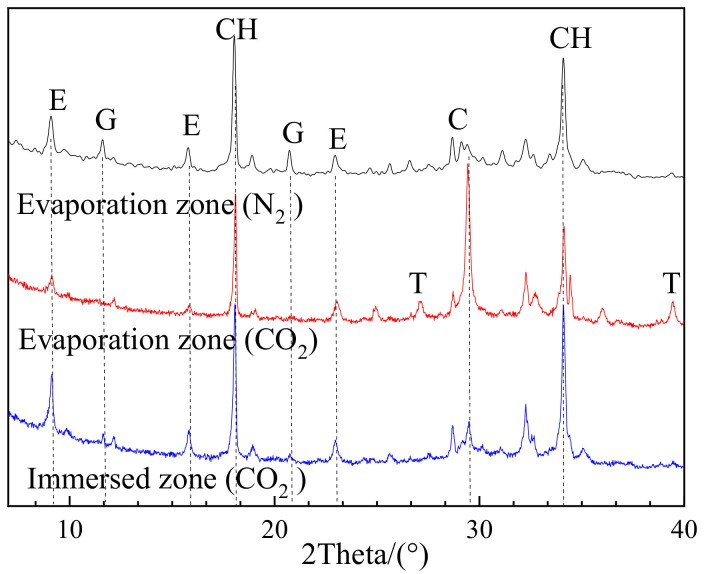
XRD patterns of PC0.45 in N_2_ and air environment after 100 days of partial exposure to sulfate attack (E—AFt; G—CaSO_4_ × 2H_2_O; CH—Ca(OH)_2_; C—CaCO_3_; T—Na_2_SO_4_).

Figure 6 presents the comparison of XRD patterns between P0.45 in N_2_ and the air environment after 100 days of partial exposure to sulfate attack. For the evaporation zone of the specimens exposed to air, a strong peak of CaCO_3_ clearly implies that the specimen is carbonated. Moreover, obvious thenardite (Na_2_SO_4_) was detected in this sample, which is a sign of the sulfate crystallization or physical attack. In contrast, the evaporation zone of the uncarbonated specimen (in N_2_ environment) has no trace of thenardite, thus there would be no physical attack. The results indicate that carbonation activates the physical attack in the evaporation zone of the cement partially exposed to sulfate attack.

On the other hand, carbonation would be able to restrain the chemical sulfate attack in the evaporation zone. In the case of uncarbonated specimens, an obvious chemical attack was found in the evaporation zone, as discussed earlier. In contrast, in the same zone of carbonated specimens, no trace of gypsum was detected, as shown in Figure 6, reflecting no chemical attack.

All these findings support the viewpoint that carbonation facilitates the physical sulfate attack, while preventing the chemical attack [15]. The suppression effect of carbonation on the chemical attack is likely because the carbonation causes the consumption of Ca(OH)_2_, resulting in insufficient Ca(OH)_2_ to chemically react with sulfates to form corrosion products, such as gypsum [12]. As a result, the crystallization of sulfates left in the evaporation zone tends to occur, and thus leads to the physical sulfate attack.

### 3.3. Thermao-Gravimetric (TG) Analysis of Sulfate Attack Products 

#### 3.3.1. Thermogravimetric Curves

**Figure 7:**
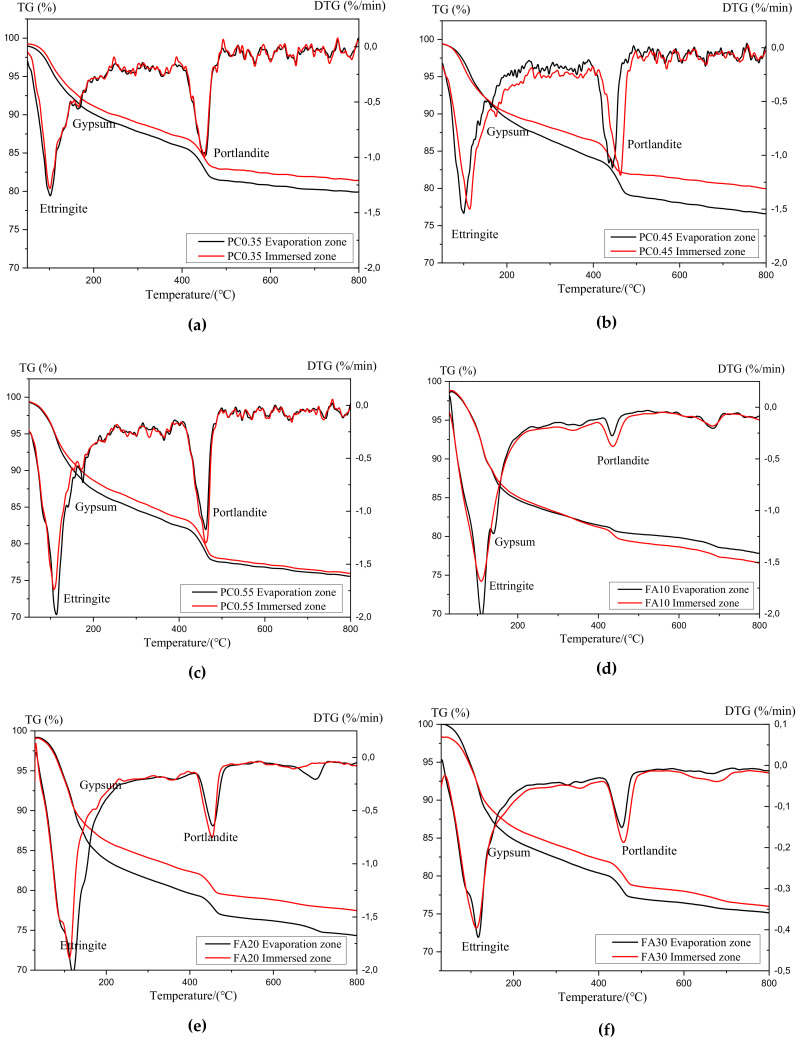
TGA and derivative thermogravimetry (DTG) curves of samples with different w/c ratios and fly ash replacement rates after 50 d exposure: (**a**) PC0.35; (**b**) PC0.45, (**c**) PC0.55, (**d**) FA10, (**e**) FA20, and (**f**) FA30.

The thermogravimetric curves for all uncarbonated samples after 50 days of the partial sulfate exposure are given in Figure 7. An obvious weight loss before 200 °C was detected for all samples, which was due to the decomposition of ettringite and gypsum [18,19,20], as well as the C-S-H gel [21,22]. The sharp peaks near 400 °C and 500 °C in the derivative thermogravimetry (DTG) curves correspond to the dehydroxylation of Ca(OH)_2_ [23]. For all mixture samples regardless of w/c ratios and fly ash replacement rates, the mass loss for Ca(OH)_2_ in the evaporation zone was higher than that in the immersion zone. Because no carbonation of specimens occurred in this work, the consumption of the Ca(OH)_2_ can only be attributed to the reaction with sodium sulfates to form gypsum. The lower content of Ca(OH)_2_ in the evaporation zone indicates more gypsum phases were formed in this zone. As one of the main products of chemical attack, gypsum can contribute to the expansion damage of the cement-based material [2,24,25,26], and a more significant amount of the expansive product tends to cause more severe deterioration. The TG analysis results demonstrated that, for uncarbonated cement paste, the deterioration in the evaporation zone still tends to be more severe than that in the immersion zone, and the deterioration mechanism is ascribed to the chemical attack.

#### 3.3.2. Quantitative Analysis of the Expansive Components

The amount of ettringite and gypsum in cement-based material tends to be overestimated using TG analysis, in which the decomposition of ettringite and gypsum is always accompanied by the decomposition C-S-H gel. To tackle this concern, a detailed interpretation of the TG analysis curve was made as follows. 

**Figure 8:**
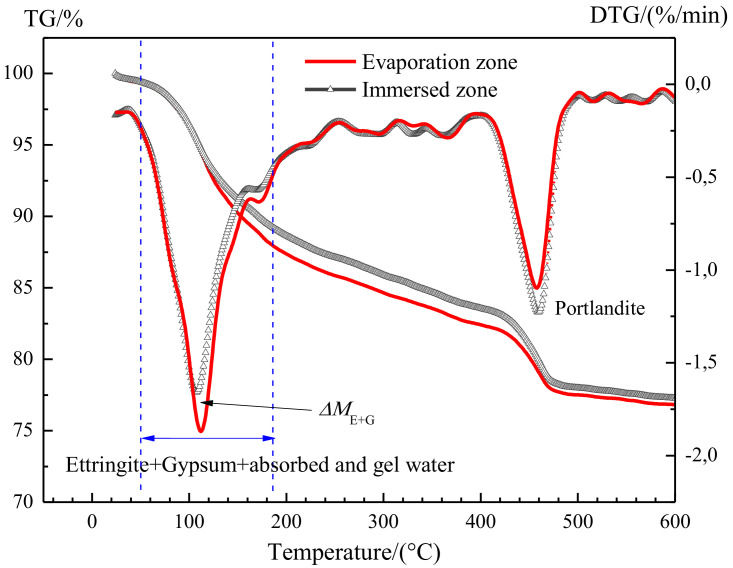
Representative TGA and DTG curves of PC0.55 at partial exposure to 5% Na_2_SO_4_ solution for 50 d.

The typical thermogravimetric curves for the uncarbonated specimen after partially sulfate exposure are given in Figure 8. A trough in the derivative thermogravimetry (DTG) curve at 50 °C to 188 °C corresponds to the decomposition of ettringite and gypsum, as well as the evaporation of absorbed and gel water in C-S-H gel:

1. The decomposition of ettringite is at 50–150 °C [18];
2. The evaporation of absorbed water is at 100–130 °C [19,21];
3. The decomposition of gypsum is at 128–188 °C [19];
4. The evaporation of gel water is at 160–185 °C [21].

Obviously, there is a partial superposition between the decomposition temperature of ettringite (or gypsum) and the evaporation temperature of absorbed water (or gel water). 

This makes it difficult to quantitively evaluate the absolute amount of ettringite and gypsum in the evaporation and immersion zone. However, the difference in the content of ettringite and gypsum between the evaporation zone and immersion zone can be evaluated by their mass loss between 50 °C and 188 °C, given that the amounts of the absorbed and gel water in evaporation zone and immersion zone are identical. This condition must have been already met in this work, because of the following:

1. Before sulfate exposure, all the specimens had been cured in saturated lime water for more than 50 days, thus cement hydration must have been nearly completed. As such, the amount of C-S-H gel in the evaporation and immersion zone should be identical and remain stable, regardless of the following-up curing regime and age.
2. During the sulfate exposure, the chemical attack of sodium sulfates would not cause the decomposition of C-S-H gel [13,27]. In fact, sodium sulfates react with Ca(OH)_2_ to form not only harmful gypsum, but also Na(OH)_2_. The resulting Na(OH)_2_ ensures the continuation of high alkalinity in the system, and thus the stability of C-S-H gel. Again, the amount of gel water in the evaporation zone and the immersion zone would remain stable.
3. After sulfate exposure, the specimens were immediately moved into a vacuum drying oven containing silica gel to remove any moisture until the start of TG analysis. Therefore, after vacuum drying, the water content in the immersion zone should not be significantly different from that in the evaporation zone.

Therefore, between 50 °C and 188 °C, the mass loss resulting from the evaporation of water in C-S-H gel should be identical in the evaporation zone and immersion zone, thus the deference in mass loss of the two zones is attributed to the different amount of ettringite and gypsum formed.

Accordingly, in this work, the mass loss at 50 °C to 188 °C was adopted for quantitative extraction of the content of gypsum and ettringite as well as absorbed and gel water, referring to *M*_E+G+W_. The difference in *M*_E+G+W_ between the evaporation zone and immersion zone was calculated and named as *ΔM*_E+G_, in order to compare the amount of ettringite and gypsum formed in the evaporation zone and immersion zone. Moreover, the content of Ca(OH)_2_ (*M*_CH_) was evaluated based on the mass loss at 400–500 °C [23], referring to *M*_CH_. The weight loss results for all samples were summarized in Table 3 and Table 4, including samples with different w/c ratios and fly ash replacement rates, after 50 days and 100 days of partial sulfate exposures. It should be mentioned that the value of *M*_E + G + W_, *M*_E + G + W_, *M*_CH_, or *ΔM*_E + G_ indicates the mass loss percentage.

As shown in Table 3 and Table 4, a positive value *ΔM_E + G_* was obtained for all the specimens, regardless of w/c ratios, fly ash replacement rates, and exposure periods. This means that the evaporation zone generated more chemical attack products and tends to suffer from more severe deterioration compared with the immersion zone. As mentioned earlier, no physical attack occurred in the evaporation and immersion zone. Therefore, the TG analysis results confirmed that chemical attack could contribute to more severe damage in the evaporation zone than in the immersion zone for cement-based materials partially exposed to sulfate attack.

#### 3.3.3. Effects of w/c Ratios

**Figure 9:**
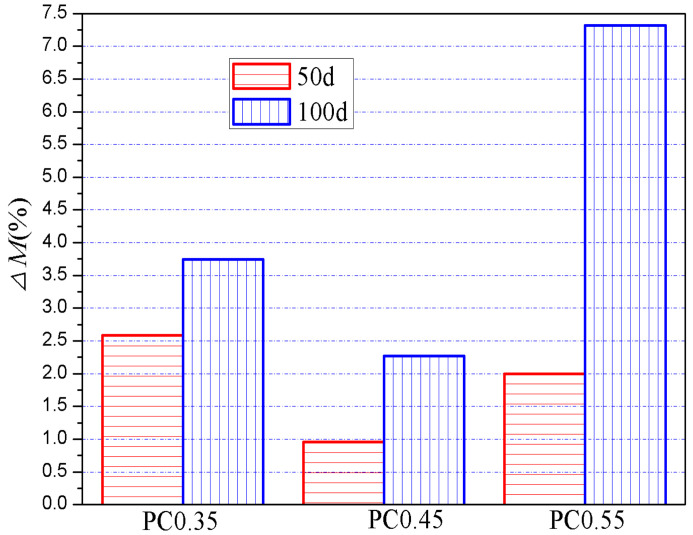
Effects of w/c on the Δ*M_E+G_* of specimens after 50-day and 100-day sulfate exposure.

Based on the data in Table 3 and Table 4, the effect of w/c ratios on the sulfate attack on all the specimens was analyzed, as shown in Figure 9. It can be seen that, with the increase in w/c ratios, the value of *ΔM_E+G_* first decreased and then increased, regardless of the exposure period. In other words, specimens with a w/c ratio of 0.45 showed the lowest difference in the chemical attack degree between evaporation and immersion zone, when compared with other w/c ratios. It worth noting that, in the XRD analysis, as shown in Figure 4, almost no chemical attack products were detected in the immersion zone of samples with the w/c ratio of 0.45, while they were detected somewhat in that of samples with w/c ratios of 0.35 or 0.55. It can be concluded, therefore, that the w/c ratio of 0.45 provides the best sulfate resistance for both the evaporation zone and immersion zone, when compared with w/c ratios of 0.35 or 0.55.

According to Figure 4, lowering the w/c ratio from 0.45 shows fewer negative effects on sulfate resistance, compared with increasing it from 0.45. For instance, the increase in the *ΔM_E + G_* when decreasing the w/c ratio from 0.45 to 0.35 was much lower than that when increasing the w/c ratio from 0.45 to 0.55, as shown in Figure 4. Considering that a lower w/c is in favor of improving mechanical performances and other durability properties because of the decrease in porosity and permeability, the w/c ratio ≤ 0.45 is recommended for cement-based materials in practice to achieve desirable overall performances.

#### 3.3.4. Effects of Fly Ash Replacements

**Figure 10:**
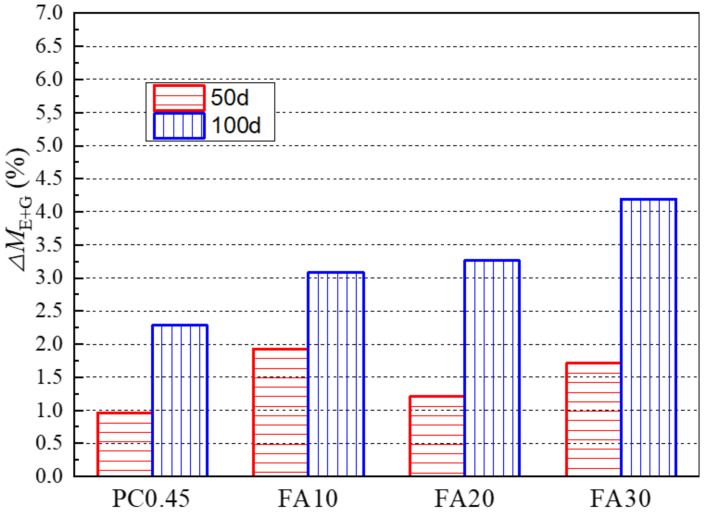
Effects of fly ash replacement rates on the *△M_E+G_* of specimens after 50-day and 100-day sulfate exposures.

The effect of fly ash replacement rates on the value of ΔM_E+G_ is given in Figure 10. For 50-day exposure, different fly ash replacement rates showed no obvious influences on the value ΔM_E+G_. However, with further sulfate exposure to 100 days, the value of ΔM_E+G_ increased significantly with the increase in fly ash replacement rates. This means that fly ash tends to cause more ettringite and gypsum formed in the evaporation zone than in the immersion zone. For the immersion zone, the XRD results mentioned earlier showed that chemical attack products such as gypsum were not detected in the immersion zone of PC 0.45, while they appeared in that of samples with fly ash. These results indicate that using fly ash as a cement replacement not only accelerates sulfate attack in the immersion zone, but also tends to promote more severe deterioration in the evaporation zone than that in the immersion zone.

These results are consistent with the work by Irassar and Najjar [4,9], while they are inconsistent with that by Chen et al. [28]. The potential reasons for the conflicting results will be discussed in the next section.

## 4. Discussion

The results of this research revealed that, for the uncarbonated cement paste partially exposed to the Na_2_SO_4_ attack, the chemical attack rather than the physical attack is the deterioration mechanism and is responsible for the more severe damage in the evaporation zone than in the immersed zone. Moreover, with the increase in w/c ratios, the sulfate attack resistance was first increased and then decreased, whereas the incorporation of fly ash caused the decrease in the sulfate resistance, especially in the evaporation zone. The potential mechanisms of the effect of the w/c ratio and the fly ash replacement are discussed as follows.

Concerning the w/c ratio, there are positive and negative effects of the w/c ratio on the sulfate resistance of the evaporation zone. On the one hand, the decrease in the w/c ratio is in favor of the decrease in the pore connectivity, hindering the penetration of the sulfate solution into the material. On the other hand, the decrease in the pore size by a lower w/c ratio tends to promote the capillary suction, resulting in more sulfates migrating into the evaporation zone. The results in this work showed that, with the decrease in the w/c, the sulfate resistance of the evaporation zone first increased and then decreased. This indicates that the positive effect of the decrease in pore connectivity dominates the sulfates resistance when the w/c ratio is higher than 0.45, resulting in a better sulfate resistance with a lower w/c. However, when the w/c is lower than 0.45, the adverse effect of the decrease in the pore size tends to neutralize and even outweigh the positive effect of the decrease in pore connectivity, thus the sulfate resistance decreases with the decrease in w/c ratios. In conclusion, the results suggest that pore connectivity dominates the sulfate resistance when the w/c ratio is higher than 0.45, whereas the pore size governs the sulfate resistance when the w/c is ≤0.45.

Concerning the fly ash replacement, there is still controversy regarding the effect of fly ash on the sulfate resistance of cement-based materials partially immersed in the sulfate-rich environment. It was reported that the fly ash addition caused a more severe deterioration of the concrete partially exposed to sulfate attack [4,9]. The authors attributed this to the fact that the decrease in pore size by fly ash tends to an increase in capillary suction height. On the other hand, Chen et al. [28] found that fly ash improved the resistance of cement mortars partially immersed in sulfate solution and ascribed this to the decrease in the connectivity of capillary pores by the continuous pozzolanic reaction. The results in this research, consistent with the results of the work by Irassar and Najjar [4,9], found the negative effect of fly ash on the sulfate resistance of the uncarbonated cement paste.

The conflicting results would be derived from the sulfate resistance being strongly influenced by the pore structures, which can be governed by the w/c ratio. There is no doubt that incorporating fly ash can reduce pore connectivity and increase the capillary sorption by reducing pore size, which has positive and adverse effects on sulfate resistance in the evaporation zone, respectively. As discussed above, the current research found that pore connectivity dominates the sulfate resistance when the w/c ratio is higher than 0.45, whereas the pore size governs the sulfate resistance when the w/c is ≤ 0.45. When the w/c is higher than 0.45, the benefit of the reduction in connectivity outweighs the adverse effect of the increase in capillary suction by the decrease in pore size, and thereby the incorporation of fly ash can improve the sulfate resistance in the evaporation zone. This could explain the results of work by Chen et al. [28], where a water-to-binder ratio of 0.65 was used. In contrast, when the w/c is ≤0.45, the adverse effect of the increase in capillary suction becomes more pronounced, and thereby the fly ash replacement can reduce the sulfate resistance. This is the case of this research, where a water-to-binder ratio of 0.45 was adopted. In contrast, when the w/c is ≤0.45, the adverse effect of the increase in capillary suction by lowering the pore size becomes more pronounced, and thereby the fly ash replacement can reduce the sulfate resistance. This is the case of this research, where a water-to-binder ratio of 0.45 was adopted.

In summary, it is suggested that the incorporation of fly ash can improve the sulfate resistance when the w/c ratio is higher than 0.45, while it reduces the sulfate resistance when the w/c ratio is equal to or lower than 0.45. The underlying reason for this phenomenon is that the decrease in pore connectivity and pore size has positive and adverse effects on sulfate resistance, respectively, and the w/c ratio can influence which one is the dominating factor. These findings highlight the significance of pore structures in how the fly ash affects sulfate attacks on cement-based materials partially exposed to sulfate attacks, which is really worth further research in the future.

## 5. Conclusions

The sulfate attack on uncarbonated cement pastes partially immersed in Na_2_SO_4_ solution was investigated, and the effects of w/c ratios and the fly ash as the cement replacement on the sulfate resistance were evaluated. The following conclusions can be drawn based on the experimental results:

1. For uncarbonated specimens after partial sulfate exposure, chemical sulfate attack products (i.e., ettringite and gypsum) were formed in both the evaporation and immersion zone, while no thenardite was identified in the evaporation zone, implying no physical attack. Moreover, more chemical attack products tend to be generated in the evaporation zone than in the immersed zone. The results confirmed that the chemical attack rather than the physical attack is the deterioration mechanism and is responsible for the more severe damage in the evaporation zone than in the immersed zone;
2. With the increase in w/c ratios, the sulfate resistance of the evaporation zone first increased and then decreased, and the best performance was achieved by a w/c ratio of 0.45. A possible reason for this phenomenon could be that the pore connectivity dominates the sulfates’ resistance of the evaporation zone when the w/c ratio is higher than 0.45, whereas the pore size governs the sulfate resistance when the w/c is relatively low (e.g., ≤0.45). To verify this viewpoint, it is recommended to further investigate the effects of the w/c on the pore structures and/or absorptivity;
3. The incorporation of fly ash resulted in a decrease in sulfate resistance of the evaporation zone. The effect of fly ash on the sulfate attack is related to the w/c used. In this study, a w/c ratio of 0.45 for the control sample was used, with which the pore size dominates the sulfate resistance performance. In this case, the pore size refinement by the fly ash replacement, leading to a higher capillary rise, can be the reason for the decrease in the sulfate resistance.

## Figures and Tables

**Table 1:** Chemical compositions of cement and fly ash (% by mass).

	CaO	SiO_2_	Fe_2_O_3_	MgO	Al_2_O_3_	SO_3_	TiO_2_	Na_2_O	K_2_O
Cement	62.68	19.62	2.961	1.89	4.37	2.06	0.236	0.11	0.711
Fly ash	3.59	53.76	6.371	0.568	31.58	0.761	1.09	0.499	1.26

**Table 2:** Mix proportions of cement paste samples. W/C, water-to-cement ratio.

Mixture Code	P·I Cement	Fly Ash	W/C	Exposure Days
PC0.35	100	-	0.35	50, 100
PC0.45	100	-	0.45	50, 100
PC0.55	100	-	0.55	50, 100
FA10%	90	10	0.45	50, 100
FA20%	80	20	0.45	50, 100
FA30%	70	30	0.45	50, 100

**Table 3:** Thermo-gravimetric (TG) analysis results of uncarbonated specimens after 50-day partial exposure (EZ—evaporation zone; IZ—immersion zone).

Specimen	Zone	M_E + G+ W_ (%)	M_CH_ (%)	ΔM_E + G_ (%)
PC0.35	EZ	7.34	9.70	2.57
	IZ	4.77	12.60	
PC0.45	EZ	5.91	8.10	0.96
	IZ	4.95	18.60	
PC0.55	EZ	10.5	7.82	1.93
	IZ	8.57	11.75	
FA10	EZ	11.11	7.02	1.21
	IZ	9.9	9.95	
FA20	EZ	11.73	7.11	1.71
	IZ	10.02	7.36	
FA30	EZ	9.94	11.90	2.00
	IZ	7.94	15.80	

**Table 4:** TG analysis results of uncarbonated specimens after 100-day partial exposure (EZ—evaporation zone; IZ—immersion zone).

Specimen	Zone	M_E + G + W_ (%)	M_CH_ (%)	ΔM_E + G_ (%)
PC0.35	EZ	7.60	7.70	3.75
	IZ	3.85	10.10	
PC0.45	EZ	6.43	7.80	2.28
	IZ	4.15	12.10	
PC0.55	EZ	12.30	5.78	3.09
	IZ	9.21	6.88	
FA10	EZ	12.12	3.81	3.26
	IZ	8.86	5.42	
FA20	EZ	13.57	3.36	4.19
	IZ	9.38	5.14	
FA30	EZ	13.57	8.10	7.32
	IZ	6.25	15.0	
